# CAF-immune cell crosstalk and its impact in immunotherapy

**DOI:** 10.1007/s00281-022-00977-x

**Published:** 2022-12-08

**Authors:** Ana Maia, Anna Schöllhorn, Juliane Schuhmacher, Cécile Gouttefangeas

**Affiliations:** 1 Institute for Cell Biology, Department of Immunology, University of Tübingen, Tübingen, Germany; 2 Cluster of Excellence iFIT (EXC2180), Image-Guided and Functionally Instructed Tumor Therapies, University of Tübingen, Tübingen, Germany; 3 SFB1399 Mechanisms of Drug Sensitivity and Resistance in Small Cell Lung Cancer, University of Cologne, Koln, Germany; 4 German Cancer Consortium (DKTK) and German Cancer Research Center (DKFZ), University of Tübingen, Tübingen, Germany

**Keywords:** Cancer-associated fibroblasts (CAFs), Tumour microenvironment (TME), T cells, Immunotherapy, Resistance

## Abstract

Tumour cells do not exist as isolated entities. Instead, they are surrounded by a variety of cells and extracellular matrix, which form the tumour microenvironment (TME). The interaction between cancer cells and their microenvironment is increasingly acknowledged as essential in dictating the outcome of the patients. The TME includes everything that surrounds tumour cells and is often highjacked by the latter to promote their growth, invasion, and immune escape. Immune cells and cancer-associated fibroblasts (CAFs) are essential components of the TME, and there is increasing evidence that their interaction constitutes a major player not only for tumour progression but also for therapy response.

Recent work in the field of immuno-oncology resulted in the development of novel therapies that aim at activating immune cells against cancer cells to eliminate them. Despite their unprecedented success, the lack of response from a large portion of patients highlights the need for further progress and improvement. To achieve its ultimate goal, the interaction between cancer cells and the TME needs to be studied in-depth to allow the targeting of mechanisms that are involved in resistance or refractoriness to therapy. Moreover, predictive and prognostic biomarkers for patient stratification are still missing. In this review, we focus on and highlight the complexity of CAFs within the TME and how their interaction, particularly with immune cells, can contribute to treatment failure. We further discuss how this crosstalk can be further dissected and which strategies are currently used to target them.

## Introduction

Cancer is the second leading cause of death in both the USA [1] and Europe (EU-27) [2], and despite new drug developments, mortality among patients remains high, with approximately 2,700,000 new cases and 1,200,000 deaths in Europe alone in 2020 [2]. It is, therefore, imperative to improve the care of cancer patients.

It is nowadays well recognised that the immune system is essential for tumour control (cancer immunosurveillance). As a result, recent years have seen a revolution in immune-based therapies against cancer. Among the different strategies, the most spectacular and promising results arose with the discovery and development of immune checkpoint inhibitors (ICIs) against CTLA-4 and PD-1 receptors [3, 4]. The approval of these antibodies by regulatory authorities has shifted the paradigm of cancer treatment towards immunotherapy by considerably enlarging awareness of the enormous potential of using the immune system to fight cancer. Even though many therapies aiming at directing the immune system to fight cancer exist, their objective response rate is rather low, their mechanisms of action are still not fully understood, and the parameters that dictate their efficacy in individual patients remain elusive. Lack of responses is now known to arise not only from tumour cell-intrinsic factors but can also be driven by the TME.

CAFs, an important component of the TME, can modulate numerous aspects of tumour biology, including therapy response. Recent studies have highlighted their heterogeneity and deepened our understanding of the functions of this cell type [5]. Their importance in regulating effective anti-tumour responses has been widely demonstrated. It is, therefore, imperative to understand the interactions between this cell type and immune cells to achieve successful outcomes in immunotherapy.

This review will give a short and general introduction to how immune cells recognise and eliminate transformed cells, which mechanisms result in the escape of cancer cells and tumour outgrowth, and which immunotherapies have been developed to date. We will then focus on describing the current knowledge in CAF biology by defining their subtypes and how these interact with different immune cell subsets. Finally, the role of the different CAF subpopulations in dictating immunotherapy outcomes and how immune cell-CAF interactions can be targeted in the clinical setting will be discussed.

## Employing the immune system to fight cancer

Despite the recent enthusiasm towards immunotherapy, the first modern attempts to use the immune system against tumour cells were done in 1891, when William Coley injected extracts of heat-inactivated bacteria (Colley’s toxins) into the tumours of cancer patients to elicit an immune attack against the tumour [6]. In 1909, Paul Ehrlich also hypothesised about the importance of immune cells in controlling tumour growth [7]. However, the potential of immune cells to control tumour progression was long defied by a series of experimental observations [8, 9]. Nevertheless, the belief that naturally arising tumours are not immunogenic was finally challenged in 1982, when researchers Aline Van Pel and Thierry Boon showed that through vaccination, immunity against spontaneous tumours could be generated [10]. Moreover, work on immunosurveillance by Lewis Thomas and Sir Frank Burnet [11] led Robert Schreiber to propose the theory of cancer immunoediting [12]. Further supporting evidence was provided by seminal works of Thierry Boon, which resulted in the identification of the first mouse [13] and human [14] tumour antigens that could be recognised by T cells. It is now well known that a crucial arm in cancer immunosurveillance is the recognition of antigens presented at the tumour cell surface by T lymphocytes [12]. Recognition and elimination of cancer cells by immune cells is a coordinated multifaceted process that requires the action of multiple cell types.

### Recognition and elimination of cancer cells by immune cells

**Fig. 1:**
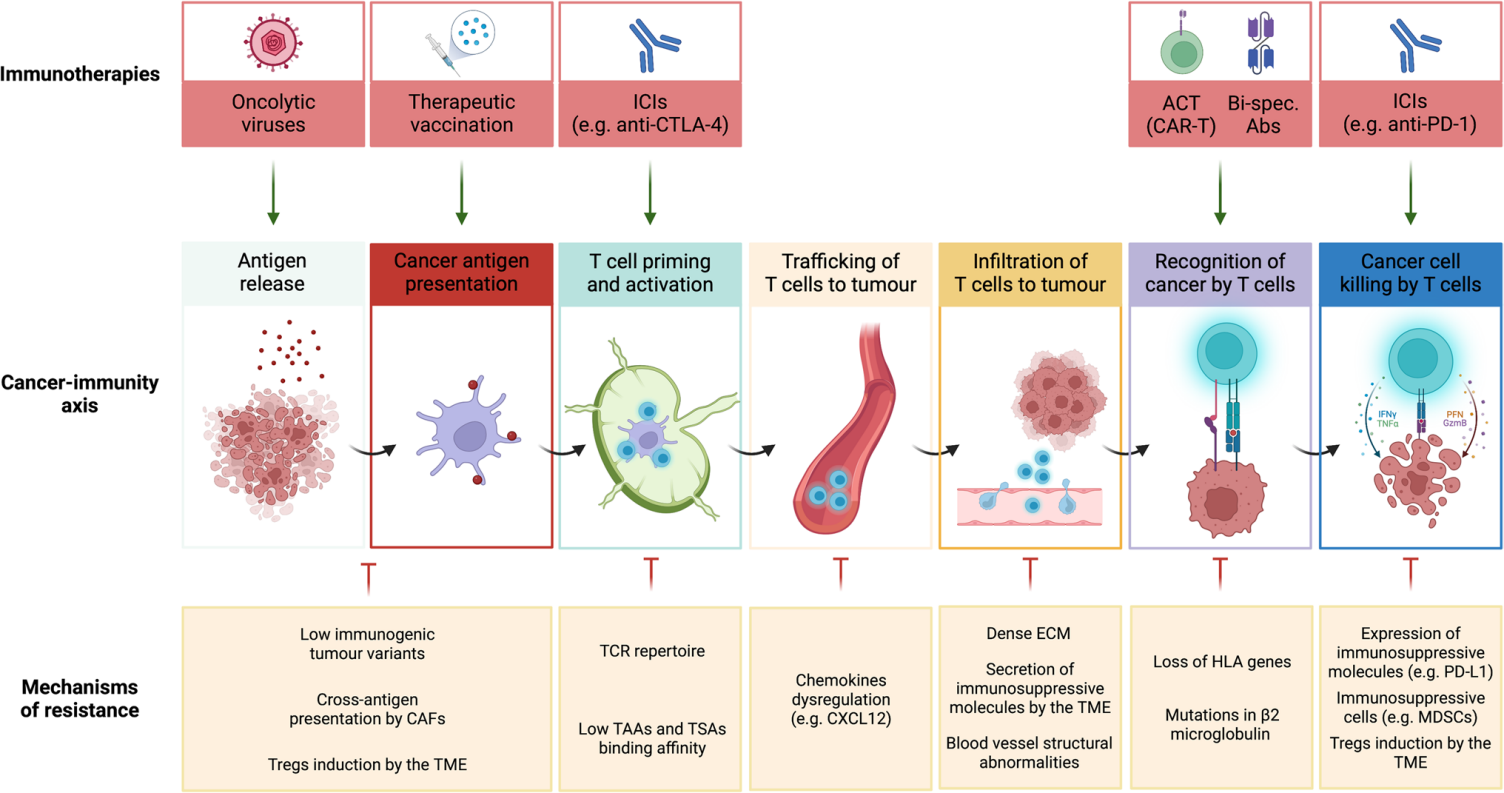
Cancer-immunity axis, mechanisms that drive its failure and impact of immunotherapies. Green arrows describe a positive correlation or effect while red arrows show an inhibitory effect. Cancer-immunity axis is adapted from [15]. Abbreviations: ICIs, immune checkpoint inhibitors; ACT, adoptive T cell transfer; Treg, regulatory T cell; TAAs, tumour-associated antigens; TSAs, tumour-specific antigens; Bi-spec. abs, bispecific antibodies; CAFs, cancer-associated fibroblast; TME, tumour microenvironment; ECM, extracellular matrix. Created with BioRender.com

CD8-expressing T cells are the ‘warriors’ of the immune system. They can recognise antigens presented at the surface of transformed cells and directly trigger a cytotoxic reaction that results in the killing of target cells. However, successful activation of these ‘warriors’ requires a full ‘army’ of immune cells from the innate and adaptive immunity arms, working in a coordinated manner. In the context of cancer, the important steps for the recognition and elimination of tumour cells by immune cells are simplified in the cancer-immunity cycle described by Chen and Mellman [15]. Very briefly, this cycle is composed of seven important steps: after cancer cell death, antigens (including tumour-associated antigens (TAAs) and tumour-specific antigens (TSAs)) are released (1) and taken up by antigen-presenting cells (APCs), especially by dendritic cells (DCs). DCs travel throughout the lymph vessels to the local lymph node, where peptides of the processed antigen are presented by HLA molecules on the cell surface of DCs to naïve T cells (2). T cells get activated after T cell priming (3), proliferate, and traffic throughout the blood vessels to the tumour site (4), where they infiltrate the TME (5). Cancer cells expressing HLA-bound antigens at their surface identical to the ones presented in step (2) to T cells are then recognised by antigen-specific T cells (6), which initiate tumour cell killing, by releasing cytotoxic granules, which results in the release of additional antigens so that the cycle starts again. All these steps must function optimally to achieve an effective anti-tumour immune response. Several mechanisms that allow cancer cells to escape recognition and elimination have been described and are summarised in Fig. 1.

To overcome the mechanisms that prevent tumour cell elimination by immune cells and to reactivate the immune system against tumours, several immunotherapies have been developed.

### Immunotherapy

A major breakthrough in the field of onco-immunotherapy was achieved with the FDA approval of the checkpoint inhibitor Ipilimumab, a monoclonal antibody against CTLA-4, in 2011 for unresectable late-stage melanoma. The fact that 22% of patients with advanced melanoma survived for three or more years in this treatment arm was staggering [16]. Inhibitory checkpoint molecules are negative regulators of T cell activation and can, therefore, dampen T cell activity. ICIs, as the name indicates, were designed to block these molecules and, in this way, release the ‘brakes’ from T cells. Since 2011, six different ICIs, all targeting the PD-1/PD-L1 signalling pathway, have been approved for the treatment of 19 different cancer types [17]. Although a broader activity is observed for PD-1 inhibitors compared to CTLA-4, with patients from a larger number of tumour entities benefiting from treatment, the outcome is still unsatisfactory.

Another category that has seen FDA approvals includes antibody-based therapies. Antibodies for the treatment of cancer can be divided into three main categories depending on their mechanisms of action: natural properties (e.g. CD20-targeting rituximab, HER2-targeting trastuzumab), engagement of cytotoxic T cells, and delivery of cytotoxic drugs [18]. Antibodies based on their natural properties have been on the market for more than 20 years, and numerous molecules are available. However, it was in 2017 that the first and only bispecific T cell engager (BiTE) antibody, binding CD19 and CD3, was approved for the treatment of patients with relapsed or refractory B-cell precursor acute lymphocytic leukaemia (ALL) [19].

In adoptive T cell therapy (ACT), autologous or allogeneic T cells are transfused to patients. Tumour-infiltrating lymphocytes (TILs) or engineered lymphocytes can be used. Treatment of metastatic melanoma patients with TILs after lymphodepletion also harboured significant success, with approximately 20% of patients still in complete remission 3 years after treatment [20]. However, this strategy is reserved for a few tumour types since it depends on the availability of fresh tumour fragments containing T cells with antitumour activity. Engineered lymphocytes can overcome this limitation. Chimeric antigen receptor (CAR) T cells hold big promise for the treatment of tumours. In this setting, T cells isolated from the patient are manipulated in vitro to express CARs bearing an immunoglobulin domain. In addition, to allow target selection, this also overcomes HLA restriction and could, in principle, overcome mechanisms of resistance related to HLA expression loss [21]. Initially described in the early 90 s, their first FDA approval was achieved in 2017 with CAR T cells directed against CD19 for ALL treatments. Despite their potential, CAR T cells still lack to show efficiency against solid tumours, and further efforts are ongoing to improve this technology, including optimising signalling to prevent exhaustion and identifying new targets [22].

Oncolytic viruses can infect and lyse tumour cells and consequently further trigger an immune response. Only a genetically modified herpes simplex virus expressing human GM-CSF has been approved so far for advanced melanoma [23].

Therapeutic cancer vaccines are designed to boost or activate tumour-specific T cells. Initially, tumour lysates and whole cells were used to immunise cancer patients in an ‘antigen-undefined’ manner. However, a more targeted approach (‘antigen defined’) is pursued nowadays, with peptides (short and/or long) and nucleic acids (DNA or mRNA) being used in cancer vaccines. Although tumour regressions are observed in some patients, these represent a very small fraction. In Europe, no approved therapeutic vaccine is available. Work to improve numerous factors of significance for the development of effective anti-cancer vaccines is ongoing. These include the identification of targets that are specifically expressed by tumour cells, including neoantigens, adjuvant development, and combination regimens, in particular with ICIs [24, 25]. Moreover, as a result of significant improvements in techniques such as next-generation sequencing (NGS) and tandem mass spectrometry (MS), which allow the fast identification of tumour-specific HLA-peptides, the field of therapeutic vaccinations is moving towards more personalised approaches, which could rapidly improve patient outcome.

All the aforementioned therapies lack broad applicability and/or effectiveness, with only a small subset of patients achieving durable responses. Efforts to understand which parameters drive therapy success or failure in individual patients or tumour entities are ongoing, and multiple studies are unravelling complex multifactorial processes involving not only cancer-intrinsic (e.g. downregulation of HLA molecules, loss of neoantigens, among others) but also cancer-extrinsic (driven by the TME, such as extracellular matrix (ECM) deposition, immunosuppressive microenvironment) [26] (Fig. 1). In a recent study, Bagaev et al. looked at available bulk sequencing data of more than 20 different cancer entities and offered evidence of the power of the TME as a general biomarker to predict response to immunotherapy, providing a rationale for using the TME landscape as a tool to stratify patients [27].

The mechanisms by which the TME, specifically CAFs, can drive immunotherapy failure will be discussed in detail in the sections below, with a focus on the impact of CAFs on the adaptive immune system.

## Cancer-associated fibroblasts (CAFs)

It was initially observed that fibroblasts in the TME behaved like reactive fibroblasts that become activated during the process of wound healing [28]. Although a universal marker that defines all fibroblasts in the TME is lacking, numerous markers are described to be expressed by activated fibroblasts in the tumours, among which the two most prominent are fibroblast-activation protein (FAP) and alpha-smooth muscle actin (αSMA) [29]. CAFs are important producers of ECM and growth factors that can directly or indirectly affect tumour cell biology and drive a variety of pro-tumourigenic processes, such as proliferation and invasion [29]. The first hints of CAF heterogeneity arose when researchers tried to eliminate CAFs from tumours and observed contrasting results in preclinical models. While depletion of FAP^+^ CAFs from the tumour stroma led to tumour regression and improved survival in mouse models of breast and colon cancers [30, 31], targeting αSMA^+^ fibroblasts or the sonic hedgehog (Shh) signalling in CAFs to reduce the fibrotic tissue around the tumour, also known as desmoplasia, resulted in accelerated tumour growth in pancreatic ductal carcinoma (PDAC) [32, 33]. Interestingly, opposite effects on immune cell composition were observed when the distinct CAF populations were eliminated, with enhanced anti-tumour immunity and an immunosuppressive environment developing when FAP^+^ or αSMA^+^ fibroblasts were targeted, respectively. It was now clear that targeting CAFs for cancer therapy would not be an easy task and that a deeper understanding of this cell population would be necessary to make any progress in this field.

CAFs have been traditionally studied using either bulk omics methods, which lack single-cell resolution, or at the single-cell level by immunohistochemistry (IHC) or flow cytometry, which only allows the investigation of a limited number of markers. Advances in single-cell technologies, among which single-cell RNA sequencing and imaging mass cytometry, provided the boosting platform that was necessary. By employing single-cell technologies, different cancer entities have been investigated, with a strong emphasis on PDAC and breast cancer (BC), likely due to their high content of desmoplasia. Among the studied tumour types, numerous subpopulations of fibroblasts have been identified. For simplicity, CAFs are often categorised into three main subpopulations, namely myofibroblasts (myCAFs), inflammatory CAFs (iCAFs), and antigen-presenting CAFs (apCAFs) (reviewed in [5]). Very briefly, myCAFs express high levels of αSMA, secrete ECM proteins in abundance, and are driven by TGFβ. iCAFs on the other hand, secrete high levels of pro-inflammatory cytokines, and their differentiation is induced by IL-1β. The latter subtype, which is often found in less abundance in the TME, is characterised by the expression of MHC class II molecules at the cell surface. The cell of origin (reviewed in [29]) and factors to which fibroblasts are exposed throughout tumour development and progression are some of the factors contributing to the high heterogeneity and plasticity observed in this cell type, which explain differences observed in composition throughout the tumour evolution and between tumour entities.

### CAF-immune cell interactions

**Fig. 2:**
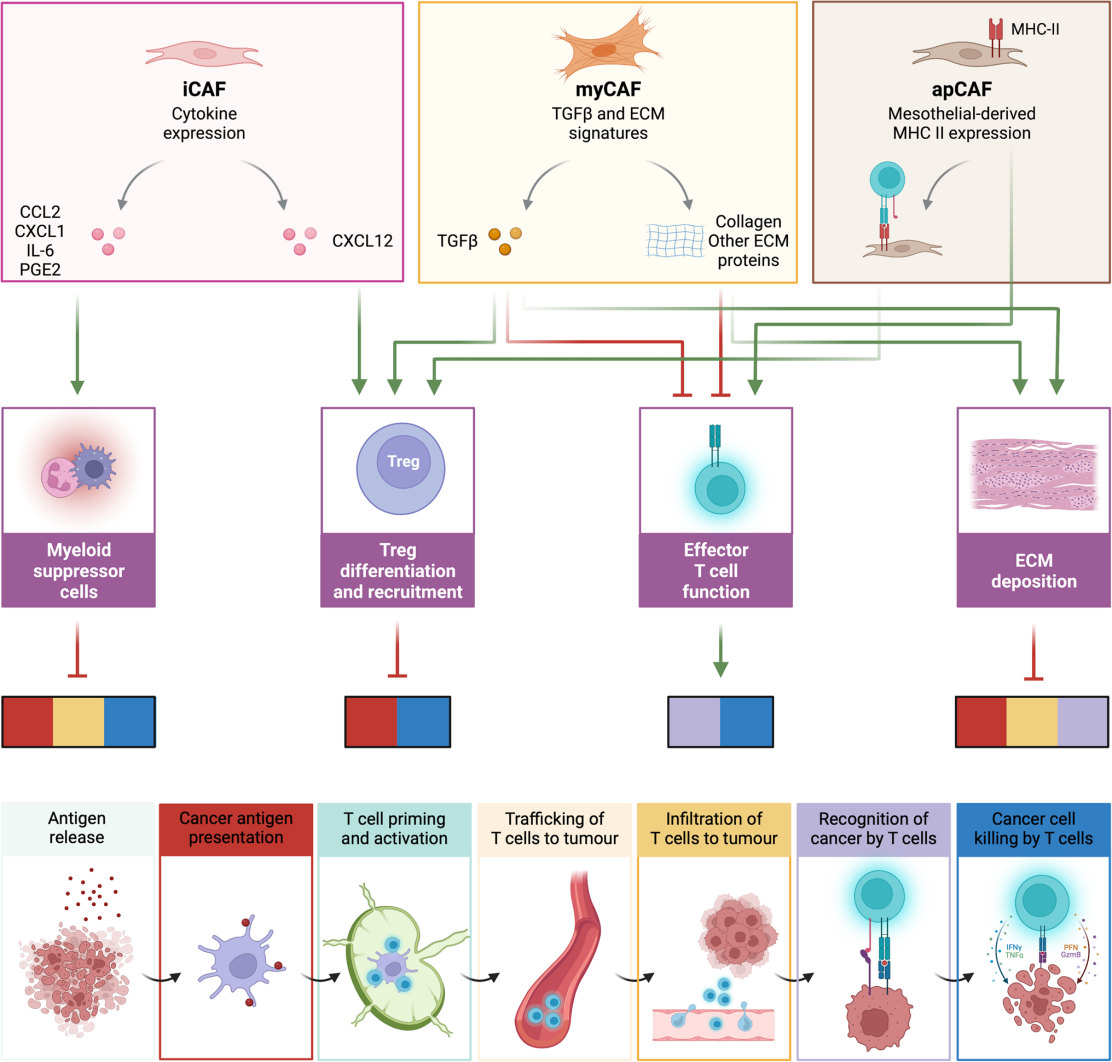
CAF subtypes, their impact on the immune milieu and on the cancer-immunity axis. Green arrows describe a positive correlation or effect while red arrows show an inhibitory effect. Colour-coded squares show the cancer-immunity axis steps which are affected by the TME components shown above. Created with BioRender.com

The mechanisms by which CAFs may alter the tumoural immune landscape are summarised in Fig. 2. Interactions between fibroblasts and immune cells that drive immunosuppression and, therefore, might contribute to the failure of immunotherapies are emphasised throughout the next sections. Nevertheless, examples in which CAFs have notable anti-tumour effects are also provided to highlight the complexity of these interactions and the difficulty of targeting CAFs for cancer treatment. Furthermore, we will mostly focus on the latest findings where heterogeneity of CAFs in the TME was investigated with single-cell technologies since their heterogeneity and in-depth study are of utmost importance.

#### CAFs and myeloid suppressor cells

CCL2, which has been shown to be secreted by CAFs but also by other cells in the TME, controls the recruitment of monocytes and myeloid-derived suppressor cells (MDSCs) [34, 35]. Interestingly, CCL2-mediated recruitment of myeloid cells was associated with resistance to checkpoint inhibition [35]. In mouse models of different tumour entities, CXCL1, which seems to be exclusively produced by iCAFs [36], promoted the infiltration of polymorphonuclear (PMN)-MDSCs into the TME and drove tumour progression [37]. Importantly, inhibition of CXCR2, the CXCL1 receptor, prevented the migration of PMN-MDSCs to the TME [37]. Selective inhibition of CXCR2 might be an interesting option since this receptor is highly expressed in CAFs and CXCL1-CXCR2 signalling controls the expression of numerous cytokines involved in the recruitment of neutrophils. Moreover, CAF-secreted IL-6, which is primarily associated with iCAFs, promotes the differentiation of myeloid cells into MDSCs in the TME [38, 39]. On the other hand, blocking of TGFβ, a molecule secreted by myCAFs, in preclinical models also resulted in a significant decrease in the amount of myeloid suppressor cells in the TME [40]. A recent study in BC using an orthotopic mouse model showed that fibroblasts in the lung metastatic microenvironment express high levels of CXCL1, IL-6, and CCL2, as well as cyclooxygenase (COX)-2 upon exposure to IL-1β. COX2^high^ CAFs secrete high amounts of prostaglandin E2 (PGE2), which induces the downregulation of molecules important for antigen presentation, including MHC-class II in DCs, and, consequently, impairs CD4^+^ and CD8^+^ T cell responses against tumour cells [41]. Moreover, several immunosuppressive genes (e.g. *Arg1*, *Ptg2*, *Nos2*, and *Il-10*) were also upregulated in DCs and other myeloid-derived cells such as monocytes upon exposure to COX2^high^ fibroblast-conditioned media. It is worth mentioning that COX2^high^ fibroblasts were present in healthy lungs and had an intrinsic immunosuppressive capacity, even in the absence of cancer. It appears that this subpopulation is more predominant in lung tissues compared to all other tissues studied. Importantly, the blockade of COX2-PGE2-EP signalling improved the efficacy of DC therapeutic vaccination as well as PD-1 inhibition [41].

#### CAFs and regulatory T cells (Tregs)

In BC, Costa et al. identified a subpopulation of fibroblasts characterised by high expression of αSMA and with immunosuppressive properties - CAF-S1. Not only do CAF-S1 secrete high levels of CXCL12, which attracted CD4^+^CD25^+^ T cells to the tumour site, but they also induced the differentiation of these cells into CD25^high^FOXP3^high^ Tregs via high expression of B7-H3, CD73, and dipeptidyl peptidase-4 (DPP4, also known as CD26) in this CAF subpopulation [42]. This goes in line with previous observations that reported a synergistic effect between the targeting of CXCL12 and PD-L1 immunotherapy in pancreatic cancer [43]. Interestingly, Elyada et al. defined CXCL12 as a marker for iCAFs rather than myCAFs in PDAC [44]. Additional dissection of CAF-S1 in BC by Kieffer et al. revealed high levels of heterogeneity within this subpopulation. Eight different CAF-S1 fibroblast clusters were defined, with some clusters actually being classified as iCAFs. The authors further show that differentiation in Tregs is mediated by a myCAF subcluster (ECM-myCAF) rather than iCAFs and that CD4^+^CD25^+^ T cells can in their turn affect the phenotype of myCAFs [45]. These findings highlight the difficulty in defining CAF subtypes and could underscore the need for more in-depth studies to understand this cell type and how it can be efficiently targeted in patients. The authors showed a correlation between the presence of ECM-myCAF cluster-specific signatures and the lack of response to PD-1 inhibitors in humans [45]. In another study, a TGFβ signature, which defines myCAFs, was also shown to associate with poor response to ICIs across several cancer types [46]. TGFβ secretion by CAFs is an important regulator of immunity, which in addition to promoting differentiation of Tregs can also directly inhibit cytotoxic T cells [47–49] and, consequently, hinder anti-tumour immunity. Furthermore, TGFβ has been shown to induce expression of PD-1 in tumours [50], and engagement of the PD-L1-PD-1 axis can, on its own, drive the formation of Tregs [51]. Indeed, targeting TGFβ in numerous models alters the immune landscape of the tumour and strongly synergises with checkpoint inhibitors [40, 52–54]. Additionally, gene expression analysis of CAFs from cancer patients shows a positive and negative correlation between myCAF-signatures and the infiltration of CTLA-4^+^CD4^+^ T cells and CD8^+^ T cells, respectively [42, 45]. It is important to note that TGFβ secretion in the TME is not exclusive from CAFs, and therefore, targeting TGFβ-secreting CAF might not be enough to deplete this molecule from the TME.

Another CAF subtype that has been shown to control Treg differentiation and promote their expansion in the TME is the apCAFs. These mesothelial-derived cells, whose differentiation has been attributed to several factors (IL-12, IFN-γ, IL-1β, and TGFβ), are characterised by the expression of MHC-class II molecules but lack expression of traditional co-stimulatory proteins (e.g. CD80, CD86, and CD40) [45, 46, 55–58]. Antigen-presentation by apCAFs in the absence of co-stimulatory molecules likely drives an anergic or regulatory state in T cells upon interaction. Interestingly, in human PDAC, the presence of apCAFs positively correlated with Tregs levels, although the authors lacked to show a link with immunotherapy outcome [55].

A recent study shows evidence that Tregs can also modulate the phenotype of CAFs in an IL-1 signalling-dependent manner [59]. The authors show that IL-1R2, a decoy receptor for IL-1β, is exclusively expressed by tumour-infiltrating Tregs in several murine and human cancer types. This results in the inhibition of IL-1β signalling through its main receptor, IL-1R1, which is mostly expressed by CAFs. Inhibition of IL-1β signalling in CAFs results in increased expression of MHC-class II, indicating that the presence IL-1R2^+^-Tregs in the TME can drive the differentiation of apCAFs, which the authors further describe, promoting the additional accumulation of Tregs. Supporting this, specific blockade of IL-1R2 in Tregs in their murine models improved anti-tumour immunity upon ICI therapy in several murine models [59].

#### CAFs and effector T cells

CAFs can express checkpoint ligands, such as PD-L1 and PD-L2, and in this way, impact T cell activation [60, 61]. Moreover, secretion of CXCL5 by CAFs in melanoma and colorectal cancer (CRC) mouse models regulates the expression of PD-L1 in tumour cells in a PI3K/AKT signalling-dependent manner [62].

In contrast to the observations reported in the previous section, a tumour-suppressive effect of apCAFs has also been described. A recent study showed a direct effect of this CAF subtype on CD4^+^ T cells, which was important to control tumour growth. In mouse models of non-small cell lung carcinoma (NSCLC), depletion of apCAFs led to accelerated tumour growth accompanied by decreased numbers of tumours infiltrating CD4^+^ and CD8^+^ T cells. apCAFs were shown to promote the survival of effector CD4^+^ T cells by inhibiting their apoptosis in a C1q-dependent manner [57]. Another interesting observation from Kerdidani et al. was that the tumour-suppressive effect of apCAFs, although observed in different models of lung cancer, could not be replicated in apCAFs derived from BC, indicating a possible tissue-dependent function of this CAF subtype [57]. A study by Hutton et al. has shown that in a PDAC mouse model, CD105 (endoglin) distinguishes two populations of CAFs with contrasting effects on immunity [58]. CD105^neg^ CAFs, which encompassed apCAFs, were able to restrict tumour growth in an adaptive immunity-dependent manner. However, the described effect was independent of the antigen-presenting capacity of apCAFs since depletion of MHC-class II, CD74, and CD80 did not abolish the tumour suppressive effect of the cells. Tumours co-injected with CD105^neg^ CAFs were more infiltrated by T and dendritic cells with higher anti-tumour response signatures compared to their CD105^pos^ counterparts. The authors further showed that these dichotomous populations of CAFs exist in human samples, although CD105 did not bear any prognostic value in human tumours [58]. The contrasting effects of apCAFs in tumour immunity could once again point to a hidden heterogeneity within this CAF subpopulation and would warrant further investigation of which mechanisms and molecules are involved in their activity prior to any attempt to target this subpopulation.

Fibroblasts from several tumour entities (lung, melanoma, and CRC) can also process and present HLA-class I peptides to CD8^+^ T cells and suppress T cell cytotoxicity through distinct mechanisms. Lakins et al. described a PD-L2 and FAS-L-induced apoptosis of T cells upon antigen cross-presentation by CAFs [61]. Although this effect was not reproduced by Harryvan et al., they observed an increase in the expression of inhibitory molecules (TIM-3, LAG3, and CD39) on the surface of CD8^+^ T cells after interaction with CAFs [63].

#### CAFs and tertiary lymphoid structures (TLS)

An interesting structure in the TME that has gained some attention in recent years is tertiary lymphoid structures (TLS). These are well-organised lymph node-like structures formed by immune cells, which can be found in non-lymphoid tissues and often develop in chronic inflammatory diseases but have been reported in certain tumours (reviewed in [64]). In the context of cancer, TLS seem to support anti-tumor immunity and are mostly associated with a favourable prognosis. Interestingly, in chronic inflammation, PDPN^+^/FAP^+^ fibroblasts are essential in the formation of these structures through a multistep process involving the secretion of numerous cytokines and chemokines (e.g. IL13, CXCL13, CCL19, and CCL21), and they also drive pathology [65]. In lung cancer, a CCL19-producing population of fibroblasts was associated with enhanced anti-tumour T cell responses and decreased tumour growth [66]. In another recent study, Rodriguez et al. showed a more direct effect of CAFs, with the fibroblast landscape determining the formation of TLS with FAP^neg^ CAFs promoting the assembly of these structures [67]. Although TME-associated fibroblasts have been implicated in the development of TLS, our understanding of this process is still bleak, and this association needs to be further addressed.

### ECM impact on the tumour immune milieu

The ECM is present in all healthy tissues, and it is composed of a complex non-cellular mesh of proteins (approx. 300 macromolecules), including collagens, glycoproteins (e.g. laminins, elastin, and fibronectin), proteoglycans (e.g. versican and hyaluran), and polysaccharides [68]. ECM biology has been consistently reported as strongly altered in the tumour context [69–71] and is often correlated with the patient outcome, with several studies throughout the years showing a prognostic value of ECM signatures in several cancer entities [72–75].

Although virtually every cell is capable of secreting ECM components, CAFs are the main architects of the ECM, with myCAFs being the main responsible for the secretion and deposition of ECM [36, 44, 45].

#### Direct impact of ECM on T cells

Immune cell-expressed inhibitory leukocyte-associated Ig-like receptor 1 (LAIR-1) has been shown to directly bind collagens in vitro [76, 77], which led to the inhibition of LAIR-1-expressing cells, including T cells. High mRNA expression of collagens, as well as LAIR-1, is associated with bad prognosis in multiple tumour types [78, 79]. Moreover, the degradation of collagen by matrix metalloprotease 1 (MMP1), which can be produced by CAFs [80], has been shown to generate LAIR-1-binding fragments. MMP1, collagen, and LAIR-1 expression were also associated with poor prognosis [79]. Importantly, collagen-driven activation of LAIR-1 has also been shown to drive CD8^+^ T cell exhaustion and dictated the response to PD-1/PD-L1 inhibition in a genetic lung cancer mouse model. In the same study, the authors showed that LAIR-1 and collagen expression in melanoma patients is predictive of ICI success, with higher levels of these markers defining poorer response to therapy and survival [81]. This goes in line with other studies that have identified ECM signatures correlated with CAF activation as markers of immunosuppression and predictors of checkpoint inhibitor response [69]. Transcriptomic analysis of T cells cultured in a 3D model revealed that high-density matrixes characterised by high collagen content drove a TGFβ-induced regulatory-like program in cytotoxic T cells while leading to the downregulation of cytotoxic markers and impairment of autologous cancer cell killing [82]. Tenascin C, another ECM protein, has also been described to inhibit the interaction between T cell-expressed integrin β1 and fibronectin, impairing T cell migration [83]. Galectins, which can be secreted by a variety of cells in the TME, including CAFs, have also been described to modulate the activity of T cells. When covered by galectin-3, TILs failed to trigger lymphocyte function-associated antigen 1 (LFA-1) and, consequently, were unable to establish a functional secretory synapse and to secrete cytokines [84]. Moreover, direct binding of galectin-3 to the TCR can prevent TCR-CD8 colocalisation in TILs and impair cytokine secretion [85].

#### ECM and immune cell exclusion

The ECM can act as a physical ‘barrier’ to drive immune exclusion [54]. T cells move along collagen matrixes using amoeboid migration. Therefore, perpendicularly oriented and densely packed collagen fibres, which are often found in the tumour periphery, can impair T cell migration. Compared to softer matrixes, T cells migrated slower in vitro when seeded in high-density collagen matrixes [86, 87]. Other in vivo studies have shown how high collagen density in the ECM prevents the migration of T cells and can trap them outside the tumour parenchyma, in the peri-tumoural areas [88–90]. In the previously mentioned study from Bagaev et al., the authors also show that two subtypes of TME-identified signatures – immune-enriched non-fibrotic (IE) and immune-enriched fibrotic (IE-F), whose main difference is the presence of CAFs, can be discriminated by the degree of T cell infiltration, with IE-F melanomas and bladder tumours having an immune excluded phenotype characteristic of ‘cold tumours’ [27]. In metastatic urothelial cancer, lack of response to PD-1 inhibition was associated with a TGFβ signature in fibroblasts, which was linked to the exclusion of CD8^+^ T cells and their entrapment in the stromal areas. This goes in line with the role of TGFβ in driving the differentiation of myCAFs, the main ECM producers. Moreover, the authors show that targeting TGFβ signalling was beneficial in a mouse model and promoted the infiltration of T cells [54]. Several others have shown similar results and implicated TGFβ signatures in resistance to ICIs in a multiplicity of cancer entities [69, 91], as well as improved T cell penetration in the tumour upon TGFβ inhibition [40, 91, 92].

In a recent study, Chen et al. showed that CAF-secreted IL-17 enhanced HIF-1α translation, which in turn promoted the expression P4Hs and LOX. Higher expression of these genes resulted in increased collagen deposition, which in turn led to the exclusion of T cells and, ultimately, caused resistance to PD-1 inhibition in cutaneous squamous cell carcinoma murine models [93]. The authors also demonstrate the prognostic value of IL-17 in human solid tumours. It is important to note that although this ‘barrier’ function of the stroma has a negative impact on immune cell infiltration to the tumour bed and traps effector T cells at the periphery, it has been previously shown that it can also work as a protective mechanism against invasion and proliferation of tumour cells [33, 94, 95]. Therefore, strategies to target the ECM need to be carefully thought out and elegantly designed.

### Targeting CAF-immune cell interactions to improve immunotherapy

Elimination of CAFs from the TME has shown not to prove an efficient therapeutic strategy, likely because of their high heterogeneity. Targeting specific signalling molecules or fibroblast subpopulations might overcome this problem, but it remains a challenge. An overview of CAF-targeting molecules that have been developed, their use in clinical trials, and their outcome are reviewed in [5, 96]. Despite efforts to target specific pathways, most of these strategies have failed to demonstrate a clear clinical benefit in humans, with most clinical trials failing before reaching phase 3 [96].

Therapies combining immunotherapies and CAF-targeting, such as described in the sections above, are also currently under investigation, with many clinical trials ongoing in numerous tumour entities. Despite the very good efficacy seen in preclinical models, results in patients are still disappointing. For example, M7824, a bifunctional fusion protein targeting both TGFβ and PD-L1, has 53 entries on www.clinicaltrial.org, with seven of these trials (NCT04501094, NCT03451773, NCT04327986, NCT04296942, NCT04428047, NCT04727541, and NCT04648826) being withdrawn or terminated early due to safety concerns or disease progression.

Preclinical models are homogeneous and often fail to reproduce human intra- and inter-tumour heterogeneity. Tumours are complex entities with an extraordinary capacity for adaption and high levels of heterogeneity. Intertumoural heterogeneity, interpatient heterogeneity and even microenvironmental heterogeneity are seen [97]. All these observations have led to a shift towards personalised medicine in oncology. However, performing in-depth omics analyses for all patients at different time points of disease and treatment is not only costly and technically challenging but also unfeasible for defining fast treatment strategies, which is essential for cancer patients. Therefore, the identification of biomarkers to predict if a patient will respond/fail to respond to a certain therapy is imperative.

#### Biomarkers for patient stratification

The definition of biomarkers that allow the stratification of patients is of utmost importance. By now, it has become clear that a single magic bullet for all cancer patients is not achievable. In preclinical models, combination regimens between CAF-targeting drugs and immunotherapies seem to be essential to achieve significant responses.

Studies that aim at defining biomarkers for patient stratification will likely play an important role in determining successful strategies for targeting the tumour stroma. Bagaev et al. defined different TME landscapes across tumours and showed specific responses to ICIs from each [27]. Another recent study in lung cancer demonstrated the importance of CAF heterogeneity in determining response to therapy, with fibroblasts isolated from certain patients providing protection against treatment and others having no impact [56]. Although this study focused on tyrosine kinase inhibitors (TKIs), it highlights the need not only to shift the paradigm in cancer treatment and account for the TME as a crucial factor in treatment outcome, but also the importance of inter-tumour heterogeneity. Such studies will be essential to pave the path for future patient stratification based also on TME, namely CAF signatures, and for optimal therapy decisions.

Finally, in addition to identifying biomarkers, it is also essential to detect these markers in the patients for their stratification and therapy decisions. Biopsies offer the best view of tumour organisation, allow the investigation of a large number of molecules, and can be used to isolate specific cell types for further expansion and study. However, they are invasive, hardly feasible for many tumour types, and they only offer a snapshot of a very limited area of the tumour. Moreover, in metastatic disease, it is almost impossible to obtain biopsies for several sites. The blood is easily accessible but lacks information on the tumour’s environment. PET tracers are an interesting approach that allows in vivo imaging of specific markers. In addition, these can be combined with antibodies, peptides, or small molecule inhibitors, which would allow the pharmacological targeting of the tumour. Tracers against FAP have been developed and showed selective tumour uptake [98, 99].

## Conclusions

Immunotherapy stands as one of the pillars of cancer treatment. Achievement of better response rates requires not only the improvement of immunotherapy strategies so that these are able to generate more potent and targeted responses against tumours, but also the identification and targeting of mechanisms that might hinder the development of these potent responses. The TME, namely CAFs, have gained significant attention in recent years as major players in determining the success of these therapies due to their strong crosstalk with immune cells. Targeting CAF-secreted factors or specific CAF subpopulations has the potential to overcome some of the observed limitations. However, an in-depth dissection and further understanding of the interactions between immune cells and CAFs are essential. Defining biomarkers for patient stratification will equally be of utmost importance to achieve good clinical responses, as it is known for all targeted therapies.
